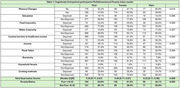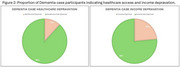# Gender Disparities in Multidimensional Poverty and Subjective Memory Complaints: Implications for Dementia Risk in Kenya

**DOI:** 10.1002/alz70860_105537

**Published:** 2025-12-23

**Authors:** Cynthia Isabel Smith, Levi A. Muyela, Jasmit Shah, Alice Moraa Ondieki, Raechel Kamau, Rachel W Maina, Anne Nyambura Njogu, Wambui Karanja, Karen Blackmon, Tamlyn J Watermeyer, Chinedu Udeh‐Momoh

**Affiliations:** ^1^ Brain and Mind Institute, Aga Khan University, Nairobi, Nairobi, Kenya; ^2^ Brain and Mind Institute, Aga Khan University, Nairobi, Kenya; ^3^ Brain and Mind Institute, Aga Khan University, Nairobi, NAIROBI, Kenya; ^4^ Female Brain Health and Endocrine Research (FemBER) Consortium, Newcastle, Edinburgh, London, United Kingdom; ^5^ University of Northumbria, Newcastle upon tyne, England, United Kingdom; ^6^ Wake Forest University, School of Medicine, Winston‐Salem, NC, USA

## Abstract

**Background:**

Alzheimer's disease and related dementias (ADRD) are rapidly increasing in Low‐and Middle‐Income Countries (LMICs), disproportionately affecting women. Beyond biological vulnerability, socioeconomic and reproductive health factors contribute to this gender disparity. Multidimensional poverty (MP)‐encompassing education, healthcare access, and living conditions‐is a known risk factor for cognitive decline, particularly among women. Additionally, subjective memory complaints (SMC), a potential early marker of dementia, are more frequently reported by women and more strongly associated to cognitive decline and dementia risk in women. This study assessed gender disparities in MP and their association with SMC in Kenya, with implications for sex‐specific dementia risk and precision health interventions.

**Method:**

Analysis utilized data from individuals recruited for brain health and dementia studies at Aga Khan University, Nairobi. The Multidimensional Poverty Index assessed deprivation across nine domains: education, food/water insecurity, barriers to healthcare, income, electricity, household assets, living standards (flush toilet access), and cooking methods. SMC was assessed via structured questionnaire ["Have you noticed a change in your memory?" yes/no]. MP was defined using a 33.33% deprivation threshold. Gender‐stratified Fisher's tests and linear regression models examined associations between poverty indicators and SMC.

**Result:**

Among 335 cognitively unimpaired participants (208 women, mean age: 53 ± 10 years) and 17 dementia cases (13 women, mean age: 71 ± 7 years), women were significantly more deprived across all poverty indicators and more frequently reported SMC. Healthcare access barriers were the most prevalent form of deprivation, disproportionately affecting women. Income deprivation was also higher in women (Table 1). Notably, none of the dementia cases were classified as multidimensionally poor, potentially indicating low health literacy, late‐stage diagnosis, and limited healthcare access among lower‐income groups.

**Conclusion:**

Gendered disparities in education, healthcare, and financial security may heighten dementia risk in women, particularly in LMICs, where early‐life socioeconomic disadvantage intersects with reproductive health challenges. These findings underscore the urgent need for targeted interventions addressing poverty‐related dementia risk factors in women, particularly access to education and reproductive healthcare. Future research will explore sex‐specific interactions between multidimensional poverty, hormonal changes, and cognitive decline, informing precision prevention strategies for ADRD in LMICs.